# Clinical whole exome sequencing revealed *de novo* heterozygous stop-gain and missense variants in the *STXBP1* gene associated with epilepsy in Saudi families

**DOI:** 10.1016/j.sjbs.2022.103309

**Published:** 2022-05-20

**Authors:** Muhammad Imran Naseer, Angham Abdulrhman Abdulkareem, Mahmood Rasool, Bader Shirah, Hussein Algahtani, Osama Y. Muthaffar, Peter Natesan Pushparaj

**Affiliations:** a Center of Excellence in Genomic Medicine Research, King Abdulaziz University, Jeddah, Saudi Arabia; b Department of Medical Laboratory Technology, Faculty of Applied Medical Sciences, King Abdulaziz University, Jeddah, Saudi Arabia; c Faculty of Science, Department of Biochemistry, King Abdulaziz University, Jeddah, Saudi Arabia; d Department of Neuroscience, King Faisal Specialist Hospital & Research Centre, Jeddah, Saudi Arabia; e King Abdulaziz Medical City, King Saud Bin Abdulaziz University for Health Sciences, Jeddah, Saudi Arabia; f Department of Pediatrics, Faculty of Medicine, King Abdulaziz University, Jeddah, Saudi Arabia

**Keywords:** Stop-Gain Mutation, Heterozygous, *STXBP1*, Developmental and Epileptic Encephalopathy 4, Saudi Arabia, STXBP1, Syntaxin-binding protein 1, EEG, Electroencephalogram, WES, Whole exom sequencing, MRI, Magnetic Resonance Imaging, DNA, Deoxyribonucleic acid

## Abstract

Intellectual disability and developmental encephalopathies are mostly linked with infant epilepsy. Epileptic encephalopathy is a term that is used to define association between developmental delay and epilepsy. Mutations in the *STXBP1* (Syntaxin-binding protein 1) gene have been previously reported in association with multiple severe early epileptic encephalopathies along with many neurodevelopmental disorders. Among the disorders produced due to any mutations in the *STXBP1* gene is developmental and epileptic encephalopathy 4 (OMIM: 612164), is an autosomal dominant neurologic disorder categorized by the onset of tonic seizures in early infancy (usually in the first months of life). In this article, we report two Saudi families one with *de novo* heterozygous stop-gain mutation c.364C > T and a novel missense c. 305C > A p.Ala102Glu in exon 5 of the *STXBP1* gene (OMIM: 602926) lead to development of epileptic encephalopathy 4. The variants identified in the current study broadened the genetic spectrum of *STXBP1* gene related with diseases, which will help to add in the literature and benefit to the studies addressing this disease in the future.

## 1 Introduction

Epileptic encephalopathy is one of the heterogeneous group of epileptic disorders including severe epilepsies characterized by several seizure types linked with the impairs cognitive and behavioral function (Berg et al., 2010). Mechanisms leading to the epileptic encephalopathy are still unknown, but it is linked to the dysfunction of neuronal network in brain (Howell et al., 2016). Epilepsy along with developmental delay in progenies could be the genetic cause and due to the increased epileptiform activity (seizures and EEG abnormalities), or may be due to combination of both factors (Trivisano and Specchio, 2020). The prognosis of the epileptic encephalopathy is generally very poor as many overlap of such types of syndromes happens, so to identify a specific electro clinical syndrome is important for treatment and informs prognosis. Epileptic encephalopathy diagnosis linked to the structural, metabolic and genetic defects of the patients have been described previously.

The *STXBP1* gene encode very important syntaxin-binding protein 1. This protein play very important part in release of the neurotransmitters through regulation of syntaxin, via transmembrane protein receptor. Any changes or mutations in the *STXBP1* gene have been previously reported in link with multiple epileptic encephalopathies along with neurodevelopmental conditions known as infantile epileptic encephalopathy-4 (Saitsu et al., 2008, Milh et al., 2011, Deprez et al., 2010, Otsuka et al., 2010, Vatta et al., 2012, Mignot et al., 2011, Campbell et al., 2012). Early infantile epileptic encephalopathy is one of the rare autosomal dominant neurological disorder leading to mild to profound intellectual disability with epilepsy. These include West syndrome, Ohtahara syndrome, and Dravet syndrome. Other non-syndromic epilepsies were also reported including atypical Rett syndrome as well as mild to severe intellectual disability having no epilepsy (Abramov et al., 2021).

All these disorders are considered by severe intellectual disability along with cerebral dysfunction as well as refractory seizures with subsequent deterioration in cognitive, sensory, and motor functions. They are usually devastating with a very poor prognosis and are often fatal (Cornet et al., 2018). Among the disorders caused by mutations in the *STXBP1* gene is developmental and epileptic encephalopathy 4 (OMIM: 612164), which is an autosomal dominant neurological condition characterized by the onset of tonic seizures in early infancy (usually in the first months of life) (Di Meglio et al., 2015).

Missense mutations in *STXBP1* gene were identified for change in the structure of the protein. It was studied that missense mutations involved in the destabilizing the STXBP1 protein, initiating degradation and aggregation and also seen that these mutations also co aggregate wild-type *STXBP1* when coexpressed (Kovačević et al., 2018, Guiberson et al., 2018, Saitsu et al., 2008, Martin et al., 2014, Chai et al., 2016;). Parkinsonian disease signs were observed in the old age patients where there was co aggregation of mutant STXBP1 protein (Chai et al., 2016, Lanoue et al., 2019). Many missense mutations in *STXBP1* have an extra influence on function. Such as hyper-secretion phenotype were seen when the P335A/L mutation showed to lead a change in *STXBP1* results in that is supposed to endorse formation of SNARE complex and causing release of neurotransmitter (Guiberson et al., 2018, Hata et al., 1993, Munch et al., 2016, Parisotto et al., 2014, Park et al., 2017, Zhu et al., 2020). Further the missense mutation L446F showed milder impact on protein expression levels were observed as compare to other missense mutations leading to gain of function phenotype related to synaptic transmission. Mutations causing the premature stop codon results in reduced protein which retain residual function based on degree of reduced protein is less stable and inclined to degradation. Any truncated STXBP1 protein can relate with *STXBP1* and other related genes, obstructing the function by destabilizing the structure with missense mutations. So far only one report showing that reduced STXBP1 protein can be detectable in the cells, and small truncations disrupt the structure of syntaxin-1 protein binding (Hata et a., 1993), explaining the importance that a complete protein of STXBP1 is essential play its role by binding its effectors.

In most cases, seizures increase in frequency and become refractory to standard anti-epileptic medications. Affected individuals have profoundly impaired psychomotor development with poor head control, limited or no ability to walk, spastic quadriplegia, and poor or absent speech (Spoto et al., 2021). In this article, we report Saudi families with a heterozygous stop-gain and a novel missense variants in exon 5 of the *STXBP1* gene.

## 2 Methods

### 2.1 Sample collection

Families with fever followed by lower limb weakness were recruited for detailed study. The pedigree was constructed after having all information from family members. Blood samples were collected in EDTA tubes from all available family members. DNA was extracted using kit methods “QIAamp genomic DNA extraction kits” following the manufacturer's protocols. Quantification of DNA was done using Nanodrop spectrophotometer (https://www.thermofisher.com/order/catalogue/product/ND-LITE-PR) and visualization was performed using CYBR Safe dye (Thermofisher, USA) by running on a 1% agarose horizontal gel electrophoresis apparatus. Informed consent was attained from the family before the start of the study. This study was permitted by the local ethics committee of CEGMR, King Abdulaziz University and complied with all the guidelines of the Helsinki Declaration of 2013.

### 2.2 Electroencephalogram (EEG) asleep

EEG was performed under standardized conditions while the patient with sedation. ECG simultaneous recording were obtained for correlation and artifact control. Eye movement were also monitored for artifact control.

### 2.3 Whole exom sequencing

Whole exom sequencing (WES) was done only for the affected member of the family to enrich regions of interest from fragmented genomic DNA using Agilent's SureSelect Human All Exon V6 Kit, as explained previously (Naseer et al., 2021). The library was sequenced on an Illumina NovaSeq6000, 150 bp PE platform to achieve an average depth of coverage of ∼100x. Typically, ∼97% of target bases are covered >10x. After WES, FASTQ files were obtained and then converted to BAM files and then BAM files were converted to variant call format (vcf), finding a total of 103,830 in case of family and 109,635 variants for family 2. These variants were used to identify mutations that may lead to the disease based on the frequency of novel/rare variants (MAF + 0.01%), function (predicted damage by polyphene/ SIFT), heterozygous, homozygous state, pathogenicity, genomic position, protein action, and previous associations with disease-related phenotype. We used several filters and bioinformatics tools to reach to the final conclusion as shown in Table 1. The following databases and *in silico* algorithms were used to annotate and assess the impact of the variant in the context of human disease: gnomAD, 1000 Genomes, ClinVar, dbSNP, OMIM, ExAC Gene Constraints, NCIB RefSeq GenesVS-SIFT, PhyloP, VS -PolyPhen2, GERP++, MaxEntScan, GeneSplicer, NNSplice, PWM Splice Predictor. Analysis was performed using the HGVS nomenclature as implemented by the VarSeq transcript annotation algorithm.

**Table 1:** Showing the details of *in silico* analysis done for this study.

**S. No**	**Online Tools**	**Pathogenicity Score for mutation in STXBP1 gene** **Family 1 c.364C>T**	**Pathogenicity Score for mutation in STXBP1 gene** **Family 2 c.305C>A**

1	Polyphen-2 (v2.2.2, released in Feb 2013)	1.0	1.0
2	MutationTaster	Disease causing	Disease causing
3	MutationAssessor 2.0	1.23	3.8
4	Phylop (phyloP46way_placental)	0.72	0.94
5	VEST3	0.82	0.97
6	CADD	28.0	34.0
7	Phastcons 1.4	1.0	1.0
8	SiPhy 0.5	16.0	17.33
9	Exome Aggregation Consortium Version 0.3.1	0.0%	0.0%
10	1000 Genomes	0.0%	0.0%
11	Diploid Internal frequency	0.0%	0.05%
12	SIFT	0.01	0.03

The following databases and *in silico* algorithms were used to annotate and assess the impact of the variant in the context of human disease: 1000 Genomes, gnomAD, ClinVar, OMIM, dbSNP, NCIB RefSeq Genes, ExAC Gene Constraints, VS-SIFT, VS -PolyPhen2, PhyloP, GERP++, GeneSplicer, MaxEntScan, NNSplice, PWM Splice Predictor. Analysis was performed using the HGVS nomenclature (https://www.hgvs.org/mutnomen) as implemented by the VarSeq transcript annotation algorithm.

The sequenced reads are aligned to GRGh37 using BWA-mem. Variants are classified and annotated using the Golden Helix VarSeq analysis workflow, which implements ACMG guidelines. An in-house bioinformatics pipeline includes base calling, alignment of reads to GRCh37/hg19 genome assembly, primary filtering of low quality reads and likely artefacts, and subsequent annotation of variants. All disease-causing variants reported in HGMD®, in ClinVar or in CentoMD® are considered, as well as all variants with a minor allele frequency (MAF) of less than 1% in the gnomAD database. The evaluation focuses on the coding exons as well as the flanking ±20 intronic bases. All relevant inheritance patterns are considered. In addition, the provided family history information and clinical data will be used to evaluate any identified variants. All identified variants will be evaluated for pathogenicity and causality. All variants associated with the patient's disease, excluding benign or likely benign variants, will be reported. Lower quality single nucleotide or deletion-insertion variants will therefore be confirmed by Sanger sequencing analysis.

### 2.4 Sanger sequencing

Further WES results validated with the Sanger sequencing technique using the targeted primers of the reported sequence variations in the *STXBP1* gene. Sanger sequencing was performed as previously explained by Naseer et al. (2020). This variation was not identified even in 100 unrelated healthy individuals in the population. In both families parents were homozygous and the proband had a *de novo* heterozygous sequence variation.

## 3 Results

### 3.1 Clinical details of the families

#### 3.1.1 Case report of family 1

**Fig. 1:**
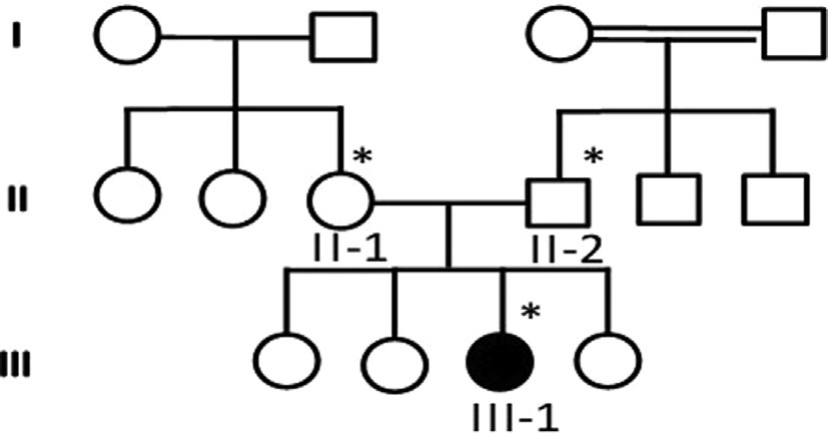
Family 1, pedigrees showing the details of all the family member participated in the study and the * sign representing the available sample for study.

**Fig. 2:**
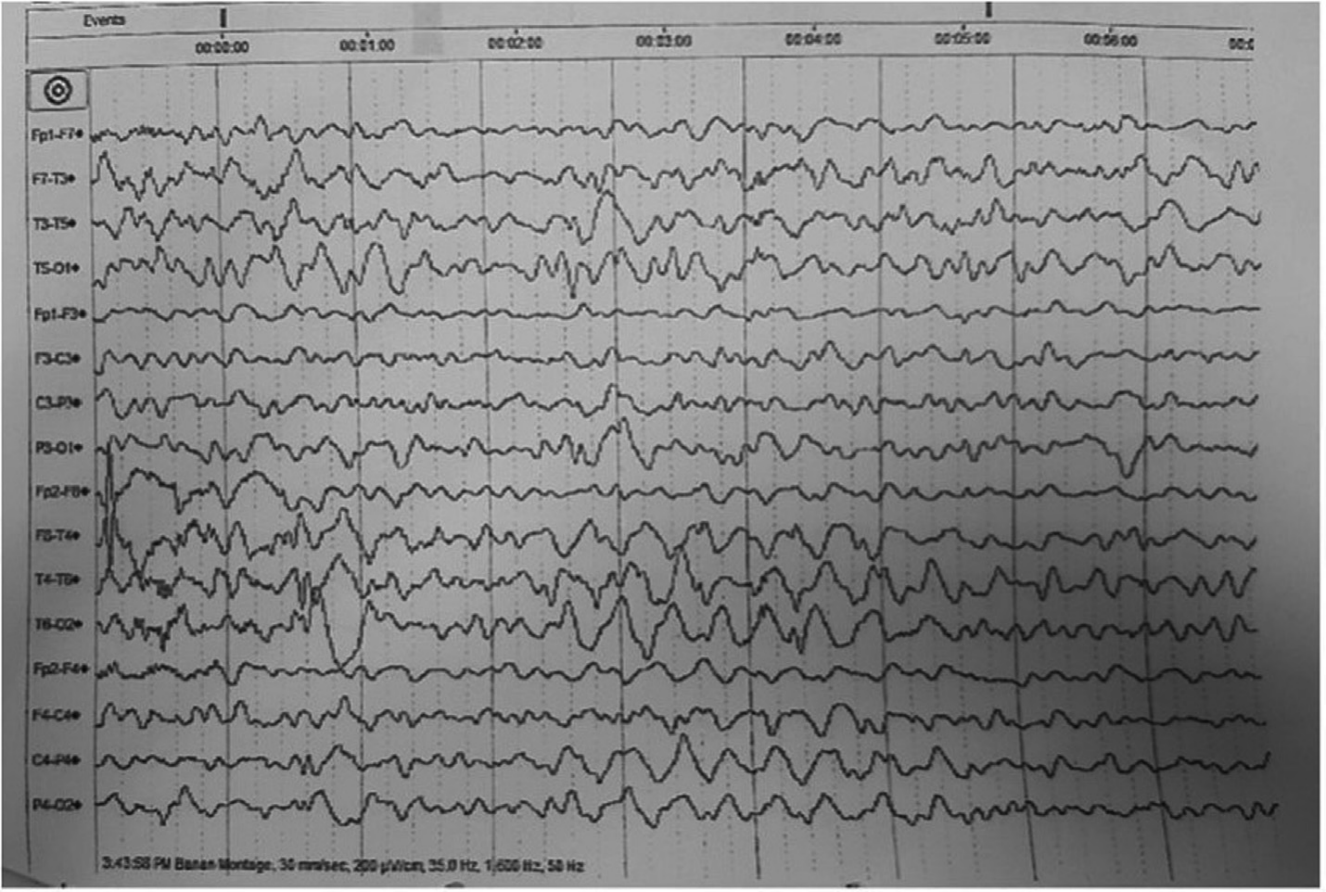
EEG showing left and right hemispheric sharp wave complexes as well as isolated sharp waves in the left temporo-occipital, left fronto-temporal, right temporo-parietal, and right fronto-temporal regions from the proband III-1 affected member of the family 1.

A 4-year-old female with a history of intractable epilepsy in the setting of global developmental delay and hypotonia. Pregnancy and labor were reported to be uneventful. The child was noted to have seizures around 6–8 weeks of age. The description of seizures given by the parents suggested multiple types of seizures including face and eyelid clonic seizures with bluish discoloration, infantile spasms (abrupt flexion and contraction of her whole body), and multifocal clonic/myoclonic seizures involving extremities. In particular, a focal motor semiology was not reported. In addition, the child has a severe global developmental delay with no development of new cognitive, language, or motor milestones. Furthermore, she has generalized hypotonia. She has two healthy older sisters and two cousins with epilepsy (Fig. 1). General physical examination showed delayed development and right hemiparesis. The remaining of her physical examination was unremarkable. Electroencephalogram (EEG) showed left and right hemispheric sharp wave complexes as well as isolated sharp waves in the left temporo-occipital, left fronto-temporal, right temporo-parietal, and right fronto-temporal regions (Fig. 2). Magnetic resonance imaging (MRI) of the brain was reported to be normal. Extensive laboratory investigations including blood and chemistry, metabolic testing, and karyotyping were unrevealing of the diagnosis. The child has been trialed with a number of anti-epileptic medications including phenobarbital, valproic acid, and topiramate without a durable seizure remission. Her final regimen consisted of levetiracetam and lamotrigine in good doses with few seizures every week to a month. The final diagnosis was developmental and epileptic encephalopathy 4.

#### 3.1.2 Case report of family 2

**Fig. 3:**
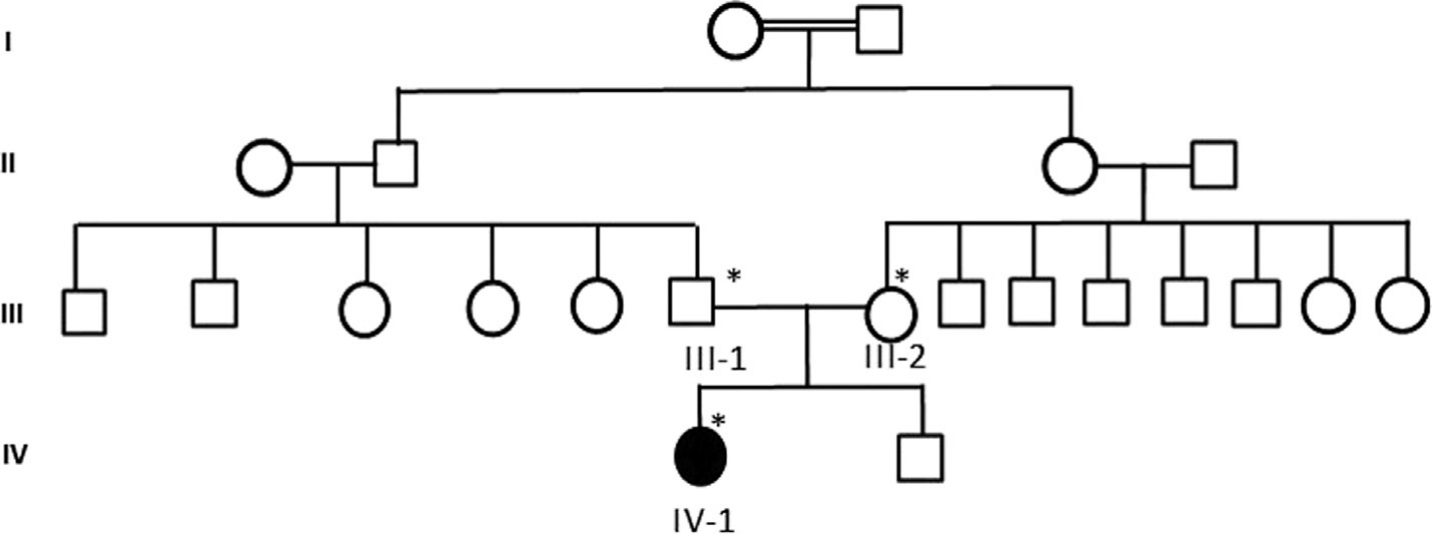
Family 2 pedigrees showing the details of all the family member participated in the study.

A four year old girl was referred to the hospital at the 2nd moth of life and was diagnosed as infantile spasm the affected member as shown in the family pedigree Fig. 3. She was started on Vigabatrin and titrated till spasm disappeared. The patient was noticed to be delayed, fully worked-up, including metabolic screen which was unremarkable. Full term pregnancy and labor were reported to be uneventful. The child was noted to have seizures around 2–3 weeks of age. Parents explained different types of seizures including face and eyelid clonic seizures with bluish discoloration, infantile spasms (abrupt flexion and contraction of her whole body), and multifocal clonic/myoclonic seizures involving extremities. In particular, a focal motor semiology was not reported. Furthermore, the child has a severe global developmental delay with no development of new cognitive, language, or motor milestones. Moreover, she has generalized hypotonia. General physical examination showed delayed development and right hemiparesis. The remaining of her physical examination was unremarkable. EEG showed excessive fast activity generalized slowing over C4, T4 and O2.

Multiplanar MRI of the brain was done without contrast. Up to date myelination for provided patient’s age. Sulci and gyri appear normal. The cerebral and cerebellar hemispheres showed normal appearances. The midline structure are intact. The midline structure were intact. Noral size ventricular system. No midline shift was present. There was no evidence of hemorrhage or spaces occupying lesion. Normal flow are seen in the cerebral arteries and in the main sinuses. Brain MRI was unremarkable. Extensive laboratory investigations including blood and chemistry, metabolic testing, and karyotyping were unrevealing of the diagnosis. The girl has been trialed with a number of anti-epileptic medications including phenobarbital and Valproic acid. Finally she was on Clonazepam 2 drops. General condition was stable. Speech Therapy along with Physical and occupational therapy was suggested. The final diagnosis was developmental and epileptic encephalopathy 4.

### 3.2 Exome sequencing

**Fig. 4:**
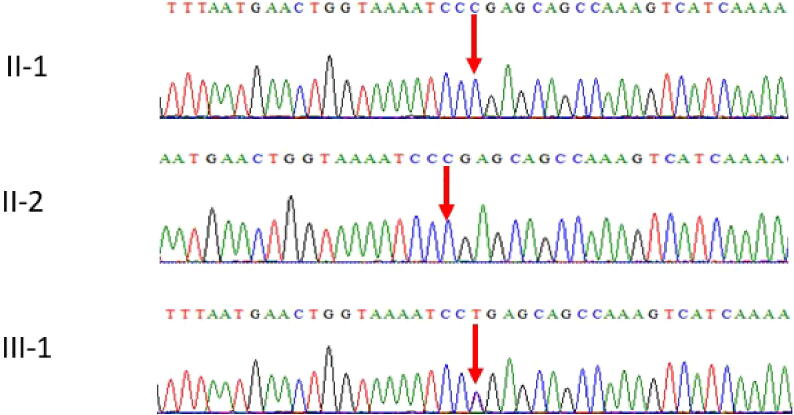
Sanger sequence analysis II-1 and II-2 are the normal wild type parents having C/C as homozygous state, while III-1 proband showing the *de novo* heterozygous stop-gain mutation c.364C > T in *STXBP1* gene.

**Fig. 5:**
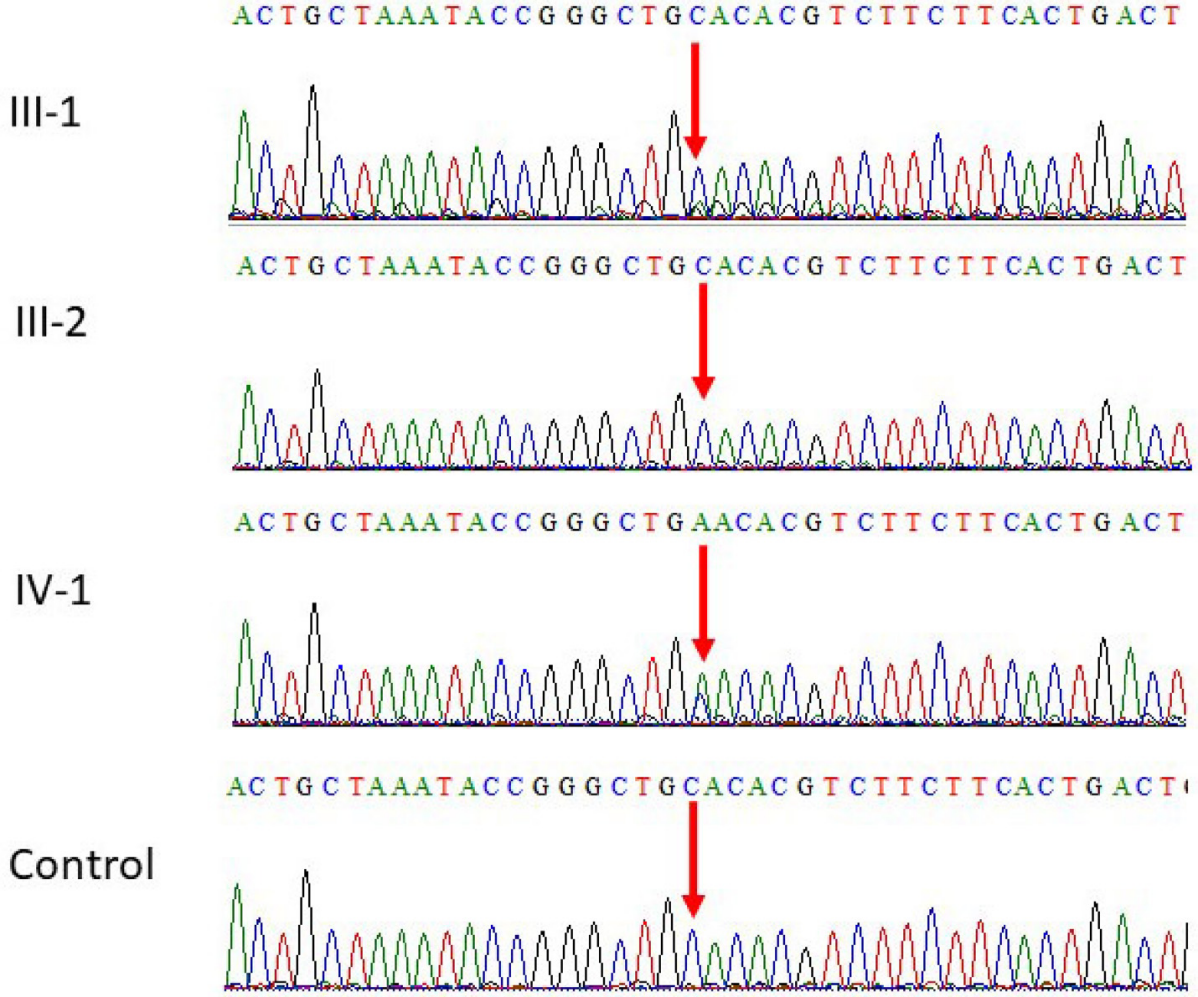
Sanger sequence analysis III-1 and III-2 are the normal parents wild type parents having C/C as homozygous state, while IV-2 proband showing a novel missense c. 305C > A in exon 5 of the *STXBP1* gene.

Clinical whole-exome sequencing analysis of the patient revealed a heterozygous stop-gain mutation in the *STXBP1* gene (OMIM: 602926). Sanger sequencing-based segregation analysis confirmed the presence of the heterozygous stop-gain mutation of c.364C > T of *STXBP1* gene in the patient (III-1) and wild type homozygous mutations in unaffected mother (II-1) and father (II-2) supporting the pathogenicity and inheritance pattern of this variant (Fig. 4). Whereas the family showed a novel missense c. 305C > A in exon 5 of the *STXBP1* gene in the affected member of the family Fig. 5.

The here detected rare variant causes a substitution of a highly conserved alanine residue into a glutamic acid at position 102. This residue is located in the hydrophobic core of STXBP1. Moreover, the detected substitution replaces the wild-type uncharged alanine with a charged glutamic acid residue, which would be predicted to severely disrupt the conformation of STXBP1. A similar suggestion has been made for other pathogenic missense mutations within the hydrophobic core of the protein (V84D, G544D and M443R) (Saitsu et al., 2008). Structural analysis of the mutant protein would be needed to verify this hypothesis. DNA analysis of the unaffected parents and the similarly affected brother is required to establish a de novo occurrence in this patient, or verify cosegregation of this variant with the phenotype. In the latter case, one of the parents is expected to be a germline mosaic for the mutation. Moreover, multiple *in silico* tools also used and they predict a harmful effect of these mutations as shown in Table 1.

### 3.3 Sanger sequencing

WES results showed *de novo* heterozygous stop-gain mutation c.364C > T and a novel missense c. 305C > A in exon 5 of the *STXBP1* gene and these results were further validated by using Sanger sequencing technique was sued to see the segregation after designing of primer for the targeted region. The results obtained after Sanger sequencing were represented in Fig. 4, Fig. 5. In the family 1 the both parents were homozygous and the proband had a *de novo* heterozygous sequence variant Fig. 4. While in family 2 the both parents were wild type normal and the proband showed novel missense mutation as shown in Fig. 5. These identified two mutations were also ruled out in 100 healthy controls samples.

## 4 Discussion

The *STXBP1* gene (also known as Munc18) having 20 exons located on the long arm of chromosome 9 at position 34.11 (Al Mehdi et al., 2019). This gene encodes a protein of the SEC1 family known as the Syntaxin1a binding protein (Stxbp1). The Stxbp1 protein is composed of 603 amino acids and is mainly expressed in the brain with involvement in the synaptic vesicle exocytosis (Kovačević et al., 2018). Mutations in this gene lead to a nonfunctional protein that eventually leads to blocking the secretion of inhibiting neurotransmitters leading to a hyperexcitation of the neurons, which consequently triggers a convulsion attack (Chen et al., 2020). Many heterozygous mutations in the STXBP1 gene include nonsense, missense, splice site mutations and frameshift along with intragenic duplications/deletions and deletion of whole gene as well. So far, no genotype and phenotype correlation of *STXBP1* has been identified and the patients are treated with different anti-epileptic drugs as the absence of any other specific therapy (Abramov et al., 2021).

Previously different types of heterozygous mutations in the *STXBP1* gene were reported including nonsense, missense, splice-site mutations, frameshift, intragenic deletions/duplications and whole gene deletions (Abramov et al., 2021). Saitsu et al., 2012, identified heterozygous missense mutations in the *STXBP1* gene in 4 unrelated Japanese patients with developmental and epileptic encephalopathy 4, three of them were de novo. Hamdan et al., 2009, identified in two unrelated French Canadian patients a *de novo* heterozygous truncating mutations in the *STXBP1* gene with severe intellectual disability and epilepsy. Furthermore, Carvill et al., 2014, identified a de novo heterozygous missense mutation in the *STXBP1* gene in an 11 year old proband with developmental and epileptic encephalopathy 4. Two additional probands with de novo heterozygous missense mutations in the *STXBP1* gene were identified using targeted resequencing of 67 patients with a similar disorder.

In this study we identified heterozygous stop-gain and novel missense variants detected in heterozygous state in the *STXBP1* gene. *STXBP1* encodes a syntaxin binding protein which play a important role in release of neurotransmitters via regulation of syntaxin, a transmembrane attachment protein receptor. STXBP1 gene mutations have been linked with epilepsy syndromes such as infantile epileptic encephalopathy type 4 (Saitsu et al., 2008, Milh et al., 2011, Deprez et al., 2010, Otsuka et al., 2010, Vatta et al., 2012, Mignot et al., 2011, Campbell et al., 2012). Early infantile epileptic encephalopathy is a rare autosomal dominant neurological disorder characterized by epilepsy and mild to profound intellectual disability. Affected newborns and infants suffer from frequent seizures, spasms, and developmental delays. Infantile spasms commonly disappear at the age 5, but in many children other types of seizures develops. Many cases of early infantile epileptic encephalopathy result from a *de novo* pathogenic mutation. Based on the study, infantile epileptic encephalopathy type 4 could explain the seizures starting from birth, and the developmental delay in this patient. *De novo* occurrence of this variant in this patient is possible. However, germline mosaic in one of the parents would be the most likely explanation in this case given that similar symptoms in the affected brother. An increased rate of germline mutations causing epilepsy have been reported in over 35 years-old fathers of epilepsy patients (Vestergaard et al., 2005).

Encephalopathies linked with *STXBP1* gene showed a extensive phenotypic spectrum, as (88%) of the patients have mild to severe type of intellectual disability while epileptic disorder was in 85% of patients which started very early in life (Stamberger et al., 2016). One-fifth of the total patients showed autism features as well (Stamberger et al., 2016). The most recurrent seizure types are epileptic spasms, motor disturbances include ataxia, dystonia, tremor, hypotonia, dyskinesia and spasticity (Stamberger et al., 2016). Moreover the awake bruxism was found in 80% of patients (Rezazadeh et al., 2019). Many old age patients having *STXBP1* gene mutations showed Parkinsonism, bradykinesia, including tremor as well as antecollis (Álvarez Bravo and Yusta Izquierdo, 2018, Keogh et al., 2015).

In one of the study they reported a total of 282 patients with *STXBP1* mutations and they established a link with their mutations types and clinical phenotypes. They identified that mainstream of these patients showed epileptic disorder that was (85%) long with developmental delay or intellectual disabilities. In relations of epileptic syndromes, most patients with *STXBP1* mutations have an unclassified (24%) of early onset epileptic (EOEE) encephalopathies and (25%) of West syndrome (Abramov et al., 2021).

The treatment of *STXBP1*-related encephalopathies is currently limited to seizure control. Several antiepileptic drugs were used including phenobarbital, valproic acid, vigabatrin, and levetiracetam. The majority of patients require the combination of more than one anti-epileptic drug. On the other hand, there is no treatment for intellectual disability and motor or behavioral disturbances (Stamberger et al., 2017).

## 5 Conclusion

In this article, we reported Saudi females child with a heterozygous stop-gain and missense variants in the exon 5 of the *STXBP1* gene leading to developmental and epileptic encephalopathy 4. The variants identified in the present study broadened the genetic spectrum of *STXBP1* gene associated with diseases, which will help to add in the literature and benefit to the studies addressing this disease in the future. Further research studies will be needed, to predict mutational effect of *STXBP1* gene, as the clinical phenotype function seems to be the same irrespective of a mutation is missense, nonsense, loss or gain of function, to improve the available therapies for the disease.

## Ethical statement

The studies involving human participants were reviewed and approved by Center of Excellence in Genomic Medicine Research Center, King Abdulaziz University Jeddah, Saudi Arabia. Written informed consent to participate in this study was provided by the participants’ legal guardian/next of kin. Written informed consent was obtained from the individual(s), and minor(s)’ legal guardian/next of kin, for the publication of any potentially identifiable images or data included in this article.

## Author contribution

AAA, and MIN designed the experiments. PNP and MIN conducted the experiments. PNP, AAA, BS and MIN analyzed the data. MR and MIN wrote the manuscript. PNP, MR and MIN finally revised the manuscript. All authors contributed to the editing of the manuscript and the scientific discussions.

## Declaration of Competing Interest

The authors declare that they have no known competing financial interests or personal relationships that could have appeared to influence the work reported in this paper.
